# Seroprevalence and risk factors for *Neospora caninum* and *Toxoplasma gondii* in dairy cattle from São Paulo State, Brazil

**DOI:** 10.1590/S1984-29612024034

**Published:** 2024-07-08

**Authors:** Juliana Correa Bernardes, Fernanda Pinto-Ferreira, Winni Alves Ladeia, Eloiza Teles Caldart, Aline Ticiani Pereira Paschoal, Thais Agostinho Martins, José Victor Pronievicz Barreto, Maria Eduarda Crespi, Luiz Daniel de Barros, Beatriz de Souza Lima Nino, Silvana Gomez Gonzalez, João Luis Garcia

**Affiliations:** 1 Universidade Estadual de Londrina – UEL, Londrina, PR, Brasil; 2 Centro Universitário de Adamantina – FAI, Adamantina, SP, Brasil

**Keywords:** Neosporosis, toxoplasmosis, dairy cattle, seroprevalence, risk factors, Neosporose, toxoplasmose, gado leiteiro, soroprevalência, fatores de risco

## Abstract

*Neospora caninum* is a major cause of reproductive loss in cattle worldwide as it leads to abortion and animal repositioning. Although *Toxoplasma gondii* does not cause a reproductive problem in cattle, consuming raw or uncooked beef poses the risk of transmission. This study aimed to evaluate the occurrence of anti-*N. caninum* and anti-*T. gondii* antibodies in dairy cattle in the West and Northwest regions of São Paulo State, Brazil. A total of 653 serum samples from dairy cows were analyzed using an indirect immunofluorescence assay (IFA). Epidemiological data from the farms were associated with the serological results of the animals by logistic regression based on the presence of antibodies. The frequencies of the antibodies against *N. caninum* and *T. gondii* were 41.6% (272/653) and 11.5% (75/653), respectively. A statistically significant association was observed between: the serum anti-*N. caninum* antibodies and breed, history of food supplementation for calves, introduction of outside animals that later presented reproductive problems, and history of reproductive problems by the trimester of gestation. The present study highlights the importance of neosporosis in dairy cattle in the study regions and that the inclusion of this parasite in the investigation of animals with reproductive disorders is important.

## Resumo

*Neospora caninum* é uma das principais causas de perda reprodutiva em bovinos em todo o mundo, pois leva ao aborto e ao reposicionamento animal. Embora o *Toxoplasma gondii* não cause problemas reprodutivos em bovinos, o consumo de carne bovina crua ou não cozida apresenta risco de transmissão. Este estudo teve como objetivo avaliar a ocorrência de anticorpos anti-*N. caninum* e anti-*T. gondii* em bovinos leiteiros nas regiões Oeste e Noroeste do Estado de São Paulo, Brasil. Um total de 653 amostras de soro de bovinos leiteiros, foi analisado utilizando-se o ensaio de imunofluorescência indireta (IFI). Os dados epidemiológicos das propriedades foram associados aos resultados sorológicos dos animais por meio de regressão logística. As frequências dos anticorpos contra *N. caninum* e *T. gondii* foram 41,6% (272/653) e 11,5% (75/653), respectivamente. Foi observada associação estatisticamente significativa entre: soropositividade para *N. caninum* e raça, histórico de suplementação alimentar para bezerros, introdução de animais externos que, posteriormente, apresentaram problemas reprodutivos e histórico de problemas reprodutivos no segundo trimestre de gestação. O presente estudo destaca a importância da neosporose em bovinos leiteiros, nas regiões de estudo, e a inclusão deste parasita na investigação de animais com distúrbios reprodutivos é fundamental.

## Introduction

Milk production is one of the major economic activities in Brazil. The country is the fifth largest milk producer in the world, with an annual yield of R$35 billion in 2019. The Southeast region is ranked the highest in milk production, accounting for 34.3% of the national yield, with 5% of the regional production from São Paulo State (Embrapa Gado de Leite, 2022).

*Neospora caninum* is a major cause of abortion in dairy cattle, and vertical transmission is considered the most relevant route (Dubey, 1999; Dubey et al, 2017; Reichel, et al., 2013). However, cattle can also be infected through horizontal transmission, which occurs through the ingestion of food or water contaminated with sporulated oocysts eliminated by the definitive hosts of the parasite, such as some species of canids (Dubey et al, 2017). In Brazil, *N. caninum* is widely distributed, with reports from 16 states (Cerqueira-Cézar et al, 2017).

Although *T. gondii* is not an important cause of abortion in cattle, it can be transmitted when the beef is consumed raw or undercooked (Santos et al., 2020). The herd can be infected through the consumption of pastures contaminated with oocysts eliminated by felines. Thus, establishing a real causal agent is important for promoting the efficiency of epidemiological knowledge and sanitation practices (Gomes et al., 2020).

The aim of this study was to investigate the occurrence of anti-*N. caninum* and anti-*T. gondii* antibodies in dairy cattle from the West and Northwest regions of São Paulo State, Brazil, and evaluate the factors associated with their seropositivity.

## Material and Methods

### Study location and ethics approval

The study was conducted on ten dairy farms in the municipalities of Tupã (four dairy farms), Iacri (one dairy farm), Queiroz (one dairy farm), Alto Alegre (two dairy farms), and Penápolis (two dairy farms), located in the West and Northwest regions of São Paulo State, Brazil. This study was approved by the Committee of Animal Ethics of the State University of Londrina (UEL) (CEUA 10/2019, number 23340.2018.90).

### Sampling

Ten dairy cattle farms with semi-intensive production systems in municipalities, where seroprevalence of *N. caninum* and *T. gondii* had not been studied were selected for this study by convenience. Blood samples were collected from Holstein, Jersey, Gir, Girolando, Jersolando, Brown Swiss, and crossbred dairy cows between March and April, 2019. The sample inclusion criteria were male and female dairy cattle up to 10 years of age. Considering the most conservative and substantial sample size obtained in epidemiological studies, an infinite population was used as a reference to calculate the sample size (n), and a prevalence of 50% was assumed for both parasites. With a 95% confidence level, the admitted error was 4%, resulting in a sample size of 600 animals.

Blood samples were collected from the herds into tubes without anticoagulants and transported in isothermal boxes at 8 °C to the Center of Veterinary Medical Diagnosis (EMERGE, Tupã, São Paulo, Brazil). The samples were centrifuged at 3000 × *g* for 10 min in the laboratory, and the serum was then transferred to a 2 mL microtube and stored at -20 °C until serological analysis.

### Serological analysis

The presence of antibodies against *N. caninum* and *T. gondii* was verified via an indirect immunofluorescence assay (IFA) using bovine anti-IgG marked with fluorescein (Fluoresceinisothiocyanate - Sigma- Aldrich, San Luis, Missouri, USA) for both parasites and slides fixed with tachyzoites from the NC-1 strain for *N. caninum* and the RH strain for *T. gondii* (Conrad et al., 1993; Camargo, 1974). One positive and one negative serum sample for antibody presence was added to each slide as a control. For *N. caninum* antibody screening, serum samples were diluted to 1:50 and 1:100. They were considered positive when calf and cow serum presented titers of ≥1:100 (Antonello et al., 2015). Similarly, for *T. gondii* antibody screening, samples were diluted to 1:16 and 1:64 and were considered positive when calf and cow serum presented titers of ≥1:64 (Santos et al., 2020). All the positive samples in the screening analysis had their titers determined by complete dilution of sera until no fluorescence reaction was observed.

### Epidemiological Characterization

To obtain epidemiological data, a questionnaire regarding information on structures and managements of farms (variables: open or close market, crossbred or pure production system), zootechnical and health characteristics of animals (variables: sex; age; breed; history of food supplementation for calves; vaccines; history of trimester in which reproductive problems occurred; and history of reproductive, neurological, previous disease, and endo-ectoparasite problems), and environment (variables: presence of dogs, presence of cats, source of water for animals, destination of wastewater, and distance between water source and wastewater) was administered.

### Statistics

Microsoft Excel® was used to tabulate the responses of producers with the variables of the epidemiological questionnaire, termed independent variables (IV), and the results of the diagnostic tests used, termed dependent variables (DV) (research results for *N. caninum* and *T. gondii* seroprevalence). Descriptive statistics were computed using Epi Info software (version 7.2.3.1).

Epi Info (version 7.2.3.1) and R environment (version 3.6.2) (epitools and epiDisplay packages) (Aragon et al, 2020; Chongsuvivatwong, 2018; Dean et al., 1996; R Core Team, 2019) were used to investigate the association between IV and DV. Significance was set at p<0.05, and the tests were chi-square corrected using Yates or Fisher's exact test or logistic regression. The strength of association was estimated using the odds ratio (OR) and its respective 95% confidence interval.

For georeferencing, the QGIS software (QGIS.org Association - Free Software Foundation, Massachusetts, New York, USA) was applied using geospatial data from the Brazilian Institute of Geography and Statistics (IBGE, 2010).

## Results

**Figure 1:**
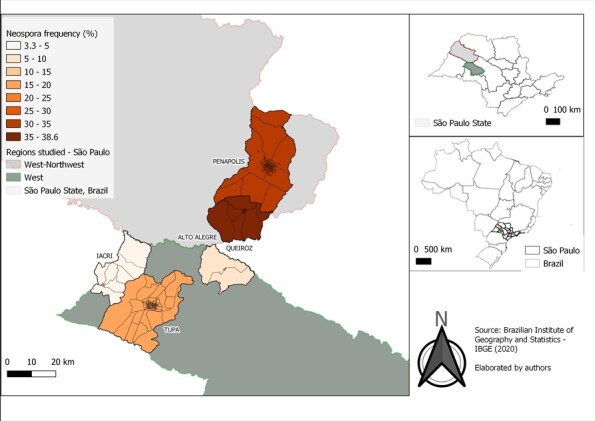
Frequency of anti-*Neospora caninum* antibodies detected via IFAT in bovine milk breeding cities in the Northwest region of Sao Paulo State, Brazil.

Blood samples were collected from 653 animals (Table 1): 251 (38.4%) in the West and 402 (61.6%) in the Northwest region of São Paulo State (Table 2). Antibodies against *N. caninum* were verified in 41.6% (272/653; CI: 37.93–45.47%) of these animals. The prevalence of protozoan antibodies was 33.5% (84/251; CI: 27.66–39.67%) in the West region and 46.8% (188/402; CI: 41.94–51.65%) in the Northwest region (Figure 1). *N. caninum* was observed in 100.00% (10/10) of the farms studied (Tables 1 and 2). Farms showed a prevalence ranging from 22.5% to 92.8%, and the range of antibody titers was 100–6,400 (Table 1 and 3).

**Table 1:** Outcome of indirect immunofluorescence assay (IFA) for IgG against *Neospora caninum* in farms from São Paulo State, Brazil.

**Farms**	**N**	**Sample (%)**	**IFA**		**Titer**	
			**pos**	**neg**	**min**	**max**
P1	26	3.98	9 (34.6%)	17	100	6,400
P2	17	2.60	9 (52.9%)	8	100	800
P3	66	10.11	19 (28.8%)	47	100	1,600
P4	40	6.13	9 (22.5%)	31	100	800
P5	72	11.03	21 (29.1%)	51	100	800
P6	173	26.49	79 (45.7%)	94	100	800
P7	30	4.59	17 (56.7%)	13	200	800
P8	110	16.85	35 (31.8%)	75	100	800
P9	91	13.94	48 (52.7%)	43	100	400
P10	28	4.29	26 (92.8%)	2	100	800
**Total**	**653**	**100.00**	**272 (41.6%)**	**381**	**100**	**6,400**

**Table 2:** Number of samples collected from cattle in West-Northwest and West regions of São Paulo State, Brazil, based on age.

**Mesoregion**	**Age of animals (months)**		
	**0-12 (%)**	**12-24 (%)**	**> 24 (%)**
West-Northwest	40/653 (6.12)	41/653 (6.28)	170/653 (26.03)
West	41/653 (6.28)	66/653 (10.11)	295/653 (45.18)
**Total**	**81/653 (12.40)**	**107/653 (16.39)**	**465/653 (71.21)**

**Table 3:** Frequency of anti-*Neospora caninum* and anti-*Toxoplasma gondii* antibodies in dairy cattle from São Paulo State, Brazil.

**Mesoregion**	***N. caninum* farms Positive/Total (%)**	***N. caninum* Animals Positive/Total (%)**	***T. gondii* farms Positive/Total (%)**	***T. gondii* Animals Positive/Total (%)**
West-Northwest	6/6 (100.0)	84/251 (33.5)	6/6 (100.0)	32/251 (12,7)
West	4/4 (100.0)	188/402 (46.8)	3/4 (75.0)	43/403 (10.7)
**Total**	**10/10 (100.0)**	**272/653 (41.6)**	**9/10 (90.0)**	**75/653 (11.5)**

Although no statistical significance was observed among age and seroprevalence of parasites, majority of the prevalence occurred in those aged ≥24 months (44.1%, 205/465).

The model obtained via multivariate logistic regression demonstrated a significant association between seropositivity for *N. caninum* and the following factors: breed, history of food supplementation for calves, introduction of outside animals that later presented reproductive problems, and history of reproductive problems during the trimester of gestation (Table 4). Herds with crossbred cows were 2.08 [(Odds-ratio Confidence Interval (OR CI): 1.30–3.31)] times more prone to be seropositive to *N. caninum* compared with purebred animals. Similarly, animals from farms without supplementation for calves were 4.67 (OR CI: 2.93–7.45) times more likely to be seropositive to *N. caninum*, and each property followed the specific diets for the calves: the main supplements used were vitamins, minerals, and protein sources. Seventy percent (7/10) of the farms practiced open market management system, in which the cows bought later presented reproductive problems. Compared to others, the chances of the cows from these farms being seropositive were 5.02 (OR CI: 3.14–8.00) times higher (Table 4).

**Table 4:** Variables statistically associated with the prevalence of anti-*Neospora caninum* antibodies in bovine milk breeding farms in the West-Northwest and West region of São Paulo State, Brazil.

**Variables**	**Positive/Total (%)**	**P***1*	**OR (CI 95%)**	**P** *2*	**Adjusted OR (CI 95%)**
**Breed**					
Crossbred	153/292 (52.4)	<0.0001	2.23 (1.62-3.07)	0.0016	2.08 (1.30-3.31)
Pure Breed	119/361 (33.0)				
**Historic of food supplementation for calves**					
Yes	209/529 (39.5)	0.0223	1.58 (1.06-2.34)	< 0.0001	4.67 (2.93-7.45)
No	63/124 (50.8)				
**Bought animals that presented later reproductive problems**					
Yes	188/402 (46.8)	0.0007	1.74 (1.25-2.42)	0.0007	5.02 (1.91-13.19)
No	84/251 (33.5)				
**Historic of reproductive problem by trimester of gestation**					
Fisrt*3*	31/112 (27.7)	0.0034		0.0007	
Second	208/468 (44.4)		1.03 (0.63-4.01)		1.12 (0.59-2.13)
Third	33/73 (45.2)		2.16 (1.16-4.01)		4.63 (4.02-4.98)

1 Bivariate; ^2^Multiple Logistic Regression; ^3^Reference Category; OR: Odds-ratio; CI: Confidence Interval.

In all the three trimesters of pregnancy, 272 (41.65%) showed a history of reproductive problems, such as abortion, retained placenta, and return to estrus. As demonstrated by the logistic model, based on the trimester of gestation, the history of reproductive problems in farms was associated with the presence of anti-*N. caninum* antibodies. Furthermore, cows with a history of reproductive problems in the third trimester of pregnancy were 4.63 times more likely to be seropositive to the protozoan compared to those with an abortion in the first trimester (Table 4). Second and third trimester of gestation had higher positivity than the first trimester of gestation (p=0.0034).

Antibodies anti-*T. gondii* was confirmed in 11.5% (75/653; CI: 9.26–14.16%) of the animals from 90% (9/10) of the farms studied (Table 3): 12.7% (32/251; CI: 8.89–17.52%) from the Northwest region and 10.7% (43/403; CI: 8.04–14.10%) from the West region. No factors were associated with the presence of *T. gondii* antibodies in the animals (Table 5).

**Table 5:** Variables statistically associated with the prevalence of anti-*Toxoplasma gondii* antibodies in bovine milk breeding farms of the West-Northwest and West region of São Paulo State, Brazil.

**Variables**	**Positive/Total (%)**	**P^1^**	**OR (CI 95%)**
**Breed**			
Crossbred	37/292 (12.7)	0.4646	0.81 (0.50-1.31)
Pure Breed	38/361 (10.5)		
**Historic of food supplementation for calves**			
Yes	6/529 (12.5)	0.1378	1.82 (0.88-3.76)
No	9/124 (7.3)		
**Bought animals that presented later reproductive problems**			
Yes	43/402 (10.7)	0.5002	1.21 (0.74-1.98)
No	32/251 (12.7)		
**Historic of reproductive problem by trimester of gestation**			
First²	15/112 (13.4)	0.2396	
Second	48/468 (10.3)		0.74 (0.40-1.37)
Third	12/73 (16.4)		1.27 (0.56-2.90)

1 Bivariate; ^2^Reference Category; OR: Odds-ratio; CI: Confidence Interval.

## Discussion

In the Northwest region of São Paulo, Curci et al. (2017) used the IFA method and observed a *N. caninum* seroprevalence of 35.1% (332/945) in dairy herds from a family farming system, and this is consistent with our results.

The seroprevalence observed for *N. caninum* in this study was 41.6%, while the seroprevalence of *N. caninum* obtained via IFA ranged from 14.1% to 97.2% in Brazil (Guimarães et al., 2004; Albuquerque et al., 2005; Ogawa et al., 2005; Benetti et al., 2009; Camillo et al., 2010; Silva et al., 2015; Chiebao et al., 2015; Klauck et al., 2016; Katto et al., 2017; Fávero et al., 2017; Snak et al, 2018; Bastos et al., 2019; Santos et al., 2020; Azevedo et al., 2021; Souza et al., 2022) and 12.4% to 43.1% in other parts of the world (Moore et al., 2002, 2009; Puray et al., 2006; González-Warleta et al., 2008; Eiras et al., 2011; Qian et al., 2017; Serrano-Martínez et al., 2007). Some of these studies used different cut-offs in their IFA, and several studies that involved the use of IFA as a serological technique have been conducted in Brazil.

Seventy percent of the studied farms had an open market management system in which the animals purchased presented reproductive problems later. These purchased animals had five times higher chances of being seropositive for *N. caninum* compared to other animals, and this is an alert to veterinarians as a control tool. All farms with crossbred animals used open market management, whereas farms with European cattle used closed herd market management. Non-replacement of animals with those from other herds can be a protective factor against *N. caninum* infection in dairy farms. Gindri et al. (2018) reported that animal purchase and animal substitution were significantly correlated with anti-*N. caninum* antibodies. A study that analyzed the impact of *N. caninum* on the reproductive parameters of 434 Holstein dairy cows from a closed herd in Brazil reported an association between the age at first calving and the services by conception, suggesting that the low seropositivity of the animals were due to closed handling system (de Barros et al., 2021).

In this study, pregnancy was observed during blood collection in 23.3% (152/653) of the cows sampled, among which 17.1% (26/152) presented history of an abortion episode that was associated with the presence of anti-*N. caninum* antibody as previously described previously (Boas et al., 2015; Bruhn et al., 2013; Chiebao et al., 2015; Corbellini et al., 2002; Hein et al., 2012; Oshiro et al., 2007; Pessoa et al., 2016). Animals in the second and third trimesters of gestation showed higher positivity for neosporosis than those in the first trimester. According to Dubey et al (2017), the pathogenesis of abortion remains unclear. Abortion caused by *Neospora* spp. occurs more frequently in the second and third trimesters of pregnancy; however, transplacental infection is very common in this gestation phase, and miscarriage rarely occurs. Nishikawa et al. (2001) and Innes et al. (2005) affirmed that a seropositive cow could experience reactivation of *N. caninum* during pregnancy, owing to the modulation of the humoral and cellular pathways of the immune system. This modulation is explained by a decrease in cellular response, which causes a change from bradyzoites to tachyzoites, thereby resulting in the invasion of the placental barrier and infection of the fetus. We observed an association between history of reproductive problems by the trimester of pregnancy and *N. caninum* seroporsitivity. Stenlund et al. (1999) and Almería et al. (2009) showed higher IgG titers in the third trimester than in the other trimesters, suggesting that the most probable period of *Neospora* reactivation is between the fourth and sixth months of pregnancy.

Even with a wide range of animal ages, no statistically significant association with *Neospora* seropositivity was observed. Some studies have revealed an association between *N. caninum* infection and animal age (Dyer et al.,2000; Guimarães et al., 2004; Sanderson et al., 2000), hypothesizing that the cause of the infection is due to the exposure time of hosts to parasite forms. Previous studies showed that younger animals had a lower seroprevalence than animals above 24 months of age. The time period for which these animals had contact with a contaminated environment probably increased their chances of infection (Bastos et al., 2019; Dyer et al., 2000; Guimarães et al., 2004).

The calves without dietary supplementation had higher susceptibility to neosporosis than the other calves in this study. Bartels et al. (1999) reported an association between farm management and the risk of infection in dairy herds in the Netherlands. According to Tokarnia et al. (2000), unequilibrated management of nutrients may occur at many levels and cause metabolic disturbances, which may decrease productivity and immunity. These may lead to reactivation of the parasite and cause clinical signs. Compared to other calves, non-supplemented calves were 4.67 times more likely to be seropositive for *N. caninum*, and they were associated with farm management because farms with the best nutrition program for cattle had the most efficient sanitation parameters as well (Chiebao et al., 2015).

This study verified the seropositivity of *T. gondii* in 11.5% of the sampled animals. Ogawa et al. (2005) observed the seroprevalence of *T. gondii* and *N. caninum* in dairy cattle from 90 farms in 12 municipalities in the northern region of Paraná and reported that 26.0% (102/392) of the animals were seropositive for *T. gondii*. In Brazil, *T. gondii* seroprevalence in cattle varies with regions and diagnostic methodology. The seroprevalence amplitude of positive animals is approximately 1%–49%, and studies have shown that the resistance of cows to *T. gondii* may interfere with prevalence rates and transmission (Albuquerque et al., 2005; Daguer et al., 2003; Dubey, 1986; Costa & Costa, 1978; Garcia et al., 1999; Marana et al, 1995). There was no association between reproductive problems and *T. gondii* antibodies, and it was expected that this parasite would not be abortifacient in cattle (Barros et al., 2021).

## Conclusion

Here, we observed a high frequency of antibodies against *N. caninum* in the studied farms; however, seropositivity was low for *T. gondii*. An association between *N. caninum* infection and history of reproductive problems was observed but not with *T. gondii*. This study shows the importance of neosporosis in dairy cattle from the study regions and including this parasite while discussing reproductive disorders in animals is fundamental for future diagnosis.
